# A Review of Electronic Early Warning Systems for Acute Kidney Injury

**DOI:** 10.1155/2024/6456411

**Published:** 2024-10-01

**Authors:** Xiangxiang Wang, Zhixiang Bian, Rui Zhu, Shunjie Chen

**Affiliations:** Department of Nephrology Shanghai Fourth People's Hospital School of Medicine Tongji University, Shanghai 200434, China

## Abstract

Acute kidney injury (AKI) is characterized by impaired renal function that can result in irreversible severe renal impairment or lifelong dependence on renal replacement therapy in some cases. Early intervention can significantly slow down the progression of AKI and reduce mortality. In recent years, electronic early warning systems for patients with AKI have been gaining attention as a potential clinical decision-support option. This paper presents a review of the application of electronic early warning systems for AKI from four aspects: development process, types of output, influencing factors, and system evaluation.

## 1. Introduction

Acute kidney injury (AKI) is a group of syndromes caused by multiple factors that lead to impaired renal function. AKI is characterized by a rapid decline in the renal function and is a common clinical emergency. Studies have shown that AKI occurs in approximately 10–15% of hospitalized patients [1]. In hospitalized patients with comorbid chronic kidney disease, the prevalence of AKI is as high as 17.6% [2], and it reaches up to 50% in patients in intensive care units [3]. Despite recent advances in renal replacement therapy, AKI is still associated with a poor prognosis, and some patients develop severe and irreversible renal impairment or lifelong dependence on renal replacement therapy. Since early intervention has shown to improve the progression of AKI and reduce mortality [4], it is important to find ways to diagnose AKI at an early stage. However, the insidious onset of AKI without typical clinical signs and symptoms may lead to delayed diagnosis or even missed diagnosis and result in progression of the disease and poor prognosis. A cross-sectional study of more than 2 million patients showed that delayed diagnosis and treatment of AKI are independent risk factors for death [5]. The Improving Global Kidney Disease Prognosis Organization (KDIGO) defines AKI as an absolute or relative increase in serum creatinine (SCr) or oliguria that lasts for 6 hours or more, as shown in Table 1. Although this definition may seem simple and straightforward, for the accurate diagnosis of AKI, it is necessary to understand baseline creatinine levels, calculate hourly urine volume/body weight, and the duration of changes in creatinine or urine volume. As a result, the diagnosis and severity staging of AKI are difficult and laborious. Independent and dedicated electronic alert systems can overcome these limitations and help detect AKI in its early stages. In fact, e-alert systems for AKI detection have been gaining popularity in recent years.

The first automatic warning system for deteriorating renal function was reported by Rind et al. as early as 1991 [6]. This system was designed to sound an alert through the computer mailbox to physicians caring for the patient when there was an increase in serum creatinine to over 0.5 mg/dl or more than 50% of the baseline value in hospitalized patients receiving nephrotoxic and renally excreted medications. Physicians were given the option to respond by indicating that the reminder was “taken care of.” This system helped physicians to adjust the dose or discontinue the drug in questions more quickly. Further in-depth studies showed that the application of the system reduced the risk of severe AKI in patients and lowered mean serum creatinine levels on days 3 and 7 [7].

**Figure 1:**
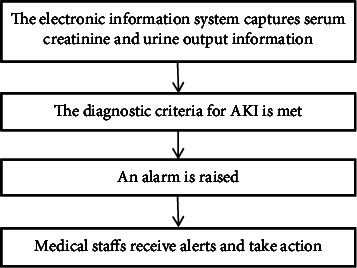
The flowchart of the electronic early warning system for AKI.

In recent years, electronic early warning systems for AKI have advanced due to the development of electronic information technology. The 2015 Acute Disease Quality Initiative Consensus Conference issued a statement that AKI early warning is an effective tool to facilitate early clinical assessment, further detection, and eventual intervention in AKI [8]. The current AKI warning systems use the patient's electronic health records or clinical information to dynamically and closely monitor changes in renal function and urine output, immediately issuing an alert when the diagnostic criteria for AKI are met. The warning facilitates a nephrology consultation or clinical decision-support system to guide the medical staff in delivering appropriate clinical interventions, thus prompting earlier clinical assessment, timely prevention, and the development of treatment strategies. The process for an AKI e-warning system is shown in Figure 1 below.

At present, there is still a lack of consensus on the application of AKI early warning systems. By combining the results and recommendations of previous studies, we will analyze the application of electronic early warning systems in AKI from four perspectives: (1) development process, (2) types of output, (3) influencing factors, and (4) system evaluation.

## 2. Development Process

Since Rind et al. [6] reported the first automatic early warning system for deterioration of AKI as early as 1991, various countries have successively developed different electronic early warning systems, as detailed in Table 2. The form of electronic warning is gradually transitioning from e-mail, telephone, and electronic medical record systems. In recent years, electronic medical record alerts have been increasingly utilized to improve the recording and identification of various diseases and medical conditions. An electronic medical record alert is a computer-based system integrated into a patient's electronic medical record. It utilizes advanced algorithms and machine learning techniques to analyze patient data and generate alerts for healthcare providers when a potential diagnosis is suspected. In AKI, the electronic medical record (EMR) alert system has the potential to analyze several factors, such as changes in serum creatinine levels, urine volume, medications, and comorbidities, to generate alerts. ACN et al. used an electronic AKI alert, which was created using the EMR system, and analyzed patient SCr values in real time following KDIGO guidelines. Due to challenges in accurately measuring the urine volume in the wards, urine output was not employed as an AKI criterion. Subsequent to the diagnosis of AKI, a noninterruptive alert displaying “Warning: probable acute kidney injury” and a link to a care bundle were issued in the patient's EMR, which can be accessed by the medical and nursing staff. The research results indicated the electronic AKI alert together with a care bundle and a multidisciplinary education program reduced the 30-day mortality rate in patients with AKI [15]. Kotwal et al. used the AKI alert bundle which includes an interruptive automated alerts in the EMR, a management guide, and primary medical staff education in the intervention group. The AKI alert bundle reduced the length of stay in most AKI patients and increased AKI records, renal disease consultation rates, and discontinuation of nephrotoxic drugs [16]. Nada and Bagwell designed an EMR-AKI alert system to trigger for neonates with SCr >1.5 mg/dL, automatically adding the AKI diagnosis to the problem list. This prompted physicians to consult nephrology, refer neonates to the nephrology clinic, and consider medication adjustments. Integration of an EMR alert system with automated documentation offers an efficient and economical solution for improving the neonatal AKI diagnosis and documentation. This approach enhances healthcare provider engagement, streamlines workflows [17]. There are several advantages for using AKI-EMR alerts with more advanced built-in automation documentation. This can help healthcare providers identify AKI in a timely manner, which is critical to initiate appropriate management and prevent related complications. In addition, EMR alerts can improve AKI diagnostic documentation in medical records, which can aid in accurate billing, data collection, and quality improvement programs, and can reduce human error and loss of important data documentation. With the maturity of machine learning technology, disease risk prediction models based on EMR and artificial intelligence technology continue to emerge. Dong et al.'s EHR data from 16,863 pediatric critical care patients between 1 month and 21 years of age from three independent institutions were used to develop a single machine learning model for early prediction of creatinine-based AKI using intelligently engineered predictors, such as creatinine rate of change, to automatically assess real-time AKI risk [18]. Predictions generate alerts allowing fast assessment and reduction of AKI risk. The model was successful in predicting stage 2/3 AKI prior to detection by conventional criteria with a median lead-time of 30 h at AUROC of 0.89. The model predicted 70% of subsequent RRT episodes, 58% of stage 2/3 episodes, and 41% of any AKI episodes. The ratio of false to true alerts of any AKI episodes was approximately one-to-one (PPV 47%). Tseng et al. utilized an artificial intelligence–based machine learning approach through perioperative data-driven learning to predict cardiac surgery–associated AKI. This study successfully established machine learning methods for predicting cardiac surgery–associated AKI, which can determine the risks after cardiac surgery, optimize postoperative treatment strategies, and minimize postoperative complications [19]. Behnoush et al. developed machine learning (ML) models to predict AKI after PCI in patients with acute coronary syndrome (ACS). Several variables were used to design five ML models: Naïve Bayes (NB), logistic regression (LR), CatBoost (CB), multilayer perception (MLP), and random forest (RF) [20]. With pre-procedural variables only, CB had the highest AUC for the prediction of AKI (AUC 0.755, 95% CI 0.713 to 0.797), while RF had the highest sensitivity (75.9%) and MLP had the highest specificity (64.35%). However, when considering preprocedural, procedural, and postprocedural features, RF outperformed other models (AUC: 0.775). In this analysis, CB achieved the highest sensitivity (82.95%) and NB had the highest specificity (82.93%). Their analyses showed that ML models can predict AKI with acceptable performance. EMR and artificial intelligence technology can conduct more research. With the maturity of machine learning technology, disease risk prediction models based on EMR and artificial intelligence technology continue to emerge. The 2019 DeepMind study developed a new deep learning algorithm by analyzing the data of 700,000 people's health electronic records, which can warn doctors 48 hours before AKI occurs, and detect AKI in 55.8% of hospitalized patients as early as 48 hours earlier [21]. However, this model dataset is mainly based on serum creatinine for the diagnosis of AKI, and serum creatinine itself is still not an ideal marker of AKI. The AKI prediction model developed by using the data of the American PCORnet platform predicted the area under the receiver operating characteristic curve (AUROC) of AKI in the internal data set was 0.84, and the AUROC in the external data set was 0.80 [22]. In contrast to the DeepMind model, the research focuses on developing interpretable “white box” AKI predictive models that can be used in different institutions and centers. The model was not validated in those patients whose length of stay was >7 days. At the same time, they used the CPT billing code to capture programs executed during hospitalization, as PCORnet CDM does not currently collect orders for programs available in real-time EMR. However, the above studies on EMR and AKI mainly focus on the establishment of retrospective prediction models, and there are few prospective studies that embed AKI risk prediction models into EMR and then intervene in high-risk populations. Future research directions of AI-based AKI early warning system include (1) combining new technologies such as natural language analysis, image pattern recognition, and incorporating more novel sensitive biomarkers to further optimize model performance; (2) the new algorithm is used to increase the interpretability of the “black box” model and provide action guidelines for clinical personalized intervention treatment; (3) targeted cluster treatment plan was designed for different high-risk groups, and clinical decision support system was built together with AKI early warning model.

## 3. Types of Output

Currently, there are three types of AKI early warning system outputs—passive early warning, blocking early warning, and active early warning—which are described below.

Passive early warning: Based on the serum creatinine and/or urine volume records, the electronic medical record system automatically issues a pop-up window to remind the occurrence of AKI. It does not interrupt the clinician's work and does not provide management advice. However, due to the controversial range of baseline values for serum creatinine, there is a possibility of false positives, as well as delayed response or lack of response. Since it does not interrupt the clinician's work, this type of output has a minimal impact on clinical behavior. A passive alert designed by Neil O'Neil et al. alerts doctors in the emergency department to abnormal values if the creatinine level exceeds the age- and gender-based creatinine standards. The study has shown that passive alert does neither improve emergency doctors' recognition of community-acquired AKI nor does they improve renal protection management [23]. Allison B McCoy et al. found the response to passive alerts about medications requiring review did not significantly change compared with baseline [9].

Blocking early warning: In the case of such outputs, the clinician needs to respond to the early warning before other actions can be performed. Compared to passive warning, it has a greater impact on clinical behavior. Kotwal S et al. reduced the length of hospital stay in most patients with AKI through an interruptive eAlert and increased the rate of renal consultation and discontinuation of nephrotoxic drugs but did not significantly improve mortality [16].

Active warning: The electronic warning system directly books nephrology consultations via telephone or in writing. It can improve the quality of the nursing process, improve patient safety, improve the effectiveness of clinical nursing, and ultimately, increase the satisfaction of providers and patients. Arwa Nada et al. used active warning can significantly improve the documentation of neonatal AKI (driven mainly by improvement of the documentation of AKI for neonates with SCr>1.5 mg/dL and referral to the neonatal nephrology clinic) and can lead to improved patient outcomes and documentation accuracy in neonatal AKI care [17].

We believe that an optimal AKI alarm system would adjust alarm dissemination according to severity levels and clinical response requirements. This form of alert closely integrates AKI detection with the suggestions offered by clinical decision support systems, ensuring that alerts are heeded by individuals who might otherwise adversely affect kidney function [24].

## 4. Influencing Factors

The main factors that influence the efficiency and application of electronic alert systems for AKI are false alerts and clinicians' attitudes towards these systems. Currently, most electronic alert systems use serum creatinine and urine volume to diagnose AKI and send out alerts. At present, most of the early warning systems are still triggered by changes in serum creatinine. With the exception of ICU wards, urine volume monitoring is difficult in other hospital settings. This can easily lead to partial or missed diagnosis and affect AKI staging. Furthermore, there is variability in the determination of baseline serum creatinine values. Therefore, using the serum creatinine at admission as the baseline could lead to missed diagnosis of AKI that occurs outside the hospital. In addition, using normal estimated serum creatinine values and ignoring the underlying chronic kidney disease basis may lead to an increase in false positives. Unlike serum creatinine records, which can be directly retrieved from the hospital's testing system, records of urine volume require manual input in most situations. Thus, urine volume records carry the risk of human error. The appearance of these false warnings in actual clinical operations may affect the attitude of doctors towards warnings, and this may reduce the effectiveness of the warning system. In addition to gathering data on changes in creatinine and urine volume, future electronic alarm systems could also be set up to detect new biomarkers. Accordingly, there has been increasing research on new biomarkers of early AKI over the last few years, and several new biomarkers have been identified, out of which we will focus on urinary kidney injury molecule 1 (KIM-1), neutrophil gelatinase-associated lipid transport protein (NGAL), tissue inhibitor of metalloproteinase (TIMP-2), insulin-like growth factor binding protein (IGFBP-7), and reactive oxygen species(RONS). These markers and their functions are described in Table 3.

### 4.1. CysC

CysC is a small molecular protein that can be freely filtered through the glomeruli and completely reabsorbed in the proximal tubules. It is almost undetectable in urine. CysC is an endogenous marker of the glomerular filtration rate. The serum CysC level is an early, sensitive, and specific predictor of AKI occurrence and an important prognostic indicator for AKI patients [25].

### 4.2. KIM-1

KIM-1 is a type I transmembrane glycoprotein and is expressed at very low levels under normal conditions. However, its expression is upregulated early in AKI, and it can be found in undifferentiated proliferating proximal tubular epithelial cells after the onset of AKI. KIM-1 is now recognized as a significant predictive marker for AKI because it is upregulated much earlier than serum creatinine and may, therefore, provide better possibilities for the diagnosis and treatment of AKI [26].

### 4.3. NGAL

NGAL is a protein with a molecular weight of 25,000 Da and is a secondary granule protein produced by neutrophils. During the process of ischemic and toxic kidney injury, the concentration of NGAL in renal tubular epithelial cells significantly increases. Within 2 h of AKI, NGAL levels in urine and serum significantly increase. Therefore, NGAL is a sensitive marker for early AKI [19].

### 4.4. TIMP-2 and IGFBP-7

TIMP-2 and IGFBP-7 are cell cycle blocking proteins expressed by renal tubular cells during cellular stress or injury and are novel biomarkers of early kidney injury [27].

### 4.5. RONS

Oxidative stress plays a major role in the early progression of AKI caused by various factors [28]. Excessive RONS lead to organelle membrane damage, DNA strand break, and protein denaturation through direct oxidation of lipids, nucleic acids, and proteins. In particular, RONS lead to an increase in the concentration of N-acetylglucosamine (NAG) and caspase-3 in turn by damaging the lysosomal membranes of the proximal tubular cells in the kidney. Last, many cell apoptosis in the kidney eventually leads to the destruction of the metabolic function of the kidney and changes of SCR and UO. According to the above analysis, the sequence of biomarker changes in the early AKI process is RONS, NAG, and caspase-3. SCR and UO only change when renal organic performance changed, which is too late to lose the value of early diagnosis. Therefore, the accurate detection of renal RONS can achieve the early diagnosis of AKI, which is essential for promptly initiating renal protective interventions to prevent the transition to more serious complications. In recent years, multiple studies have shown that nanodrugs alleviate acute kidney injury by manipulating RONS at the kidney [29].

At the same time, future electronic alarm systems could incorporate individual information such as patient risk factors, susceptibility, and exposure level (such as current medication and hemodynamic data such as hypotension records, comorbidities, historical clinical data, and admission data) to EMR to identify patients who may be at risk of kidney injury. These developments could eventually reduce the incidence of false positives. Clinicians' attitudes toward electronic warning systems will affect the clinical value of these systems. In particular, with the development of information technology and its increasing application in the clinical context, early warning fatigue among clinicians is a matter of concern. With the development of information technology, many more warning systems may be developed for use in the clinic, and this could lead to a high incidence of false positive alarms. This may cause “alarm fatigue,” and doctors could overlook all alarms, including correct alarm information, thus causing harm to patients. Secondly, as AKI is multifactorial and its causative factors can be divided into prerenal, renal, and postrenal factors, simple alerts about the occurrence of AKI without corrective suggestions based on the cause greatly reduces the value of the alarm system for many healthcare professionals.

## 5. System Evaluation

Current system evaluations of AKI electronic warning systems in clinical applications are mixed, with widely varying conclusions. More based on the AKI electronic early warning system can effectively early warning AKI, and whether it can reduce the length of hospital stay, reduce mortality and dialysis rates, and avoid acute kidney injury into chronic kidney disease, there is no unified standard. An early study covering 1518 individuals showed that the electronic warning system was able to identify AKI early on and may lead to an improved prognosis [30]. In contrast, in a study that included 6,030 adult inpatients with AKI, it was found that the EMR system did not reduce the risk of primary regression of AKI, i.e., dialysis or death [31]. In a study conducted in the intensive care unit of Mayo Hospital, the tool analyzes any rise in the serum creatinine level or decrease in urine output using the Acute Kidney Injury Network criteria by 2 independent investigators reviewed as the reference standard. The sensitivity and specificity of the AKI early warning system was found to be 88% and 96%, respectively [32]. In a prospective, randomized, controlled study conducted in the high-risk ward of Guangdong Provincial People's Hospital in China, the e-alert system which diagnosed AKI automatically based on serum creatinine levels was found to be a reliable tool for an accurate diagnosis of AKI compared by “researcher-confirmed AKI” according to 2012 KDIGO-AKI guidelines, with a sensitivity and accuracy of 99.8% and 98.1%, respectively, for the AKI e-alert system [12]. A prospective validation to harness the true clinical benefit is warranted.

## 6. Conclusion

As previously mentioned, there is no consensus about the optimal detection algorithm for AKI eAlert systems, and the current criteria used in daily practice may need to be re-evaluated. The sensitivity and specificity of each system need to be measured, and the relationship of the system to the process of care and patient outcomes needs to be examined. Because the systems currently used are so diverse in design, it is also important to assess corresponding changes in the process of care. In addition, it is necessary to check for AKI documentation, additional testing, and appropriate interventions, such as discontinuation of nephrotoxic medications, after each alarm activation. Finally, user acceptance of the system is an important parameter to be considered in the application of such electronic warning systems.

In conclusion, AKI is a common clinical emergency with a poor prognosis, and early identification of AKI by electronic warning systems and early intervention are key tools in improving the prognosis of AKI. In the future, artificial intelligence will be used to comprehensively extract patients' serum creatinine and clinical information, including patients' age, gender, underlying diseases and drug use, and combined with the use of new markers, to build a more optimized AKI model and continue to monitor relevant indicators for better clinical services. In addition to relying on these systems, clinical practitioners need to play an active role in identifying risk factors and susceptibility factors for AKI and administer early interventions to avoid serious disease progression, thus improving the overall management of AKI.

## Figures and Tables

**Table 1:** KDIGO staging of AKI.

The staging	SCr	Urine output
Stage l	1.5- to 1.9-fold increase in SCr from the baseline or ≥0.3 mg/dL (≥26.5 *μ*mol/L)	<0.5 mL/kg per hour for >6 h
Stage 2	2- to 2.9-fold increase in SCr from the baseline	<0.5 mL/kg per hour for >12 h
Stage 3	≥3-fold increase in SCr from baseline, or increase in SCr to ≥4.0 mg/dL (≥353.6 *μ*mol/L), or initiation of RRT(renal replacement therapy),or in patients <18 years old, decrease in estimated GFR (glomerular filtration rate) to <35 mL/min/1.73 m^3^	<0.3 mL/kg per hour for 24 h or anuria for 12 h

**Table 2:** The development process of electronic early warning systems.

Number	Year	Study	Country	Study subjects	Study design	Diagnostic criteria for AKI	Type of the alert	Outcomes
1	1991	Rind et al. [6]	USA	10,076 patients	A prospective time-series study	An increase in SCr level of 0.5 mg/dL or more occurred when the patient was receiving a nephrotoxic medication, or an increase in SCr level of 50% or more to 2.0 mg/dL or higher was noted during the administration of a renally excreted medication	The computer mailbox	The average time for adjusting or discontinuing medication was 21.1 hours in advance and the computerized reminders had a strong effect on physician behavior
2	2010	McCoy et al. [9]	USA	1,598 adult inpatients	Quality improvement report	A minimum 0.5 mg/dL increase in SCr level	The alert text appeared within the computerized provider order entry interface and on printed rounding reports	The rate and timeliness of modification or discontinuation of targeted drugs increased
3	2012	Colpaert et al. [10]	Belgium	951 patients	Prospective intervention study	The RIFLE classification	The call	The number and timeliness of early treatment interventions increased
4	2017	Holmes et al. [11]	UK	21 093 patients	A prospective national cohort study	KDIGO criteria	The Wales Laboratory Information Management System	The implementation of AKI e-alerts in primary care demonstrated its clinical utility
5	2018	Wu et al. [12]	China	5308 patients	A prospective, randomized, controlled study	KDIGO criteria	The e-alert system	The e-alert system was a reliable tool for accurately diagnosing AKI
6	2019	Gómez et al. [13]	Spain, USA, Italy	1079 patients	A single-center retrospective study	KDIGO criteria	The Email	It identified half of AKI episodes
7	2021	Holmes et al. [14]	UK	132 599 patients	A prospective national cohort study	KDIGO criteria	The system of AKI e-alerts	Introduction of alerts improved patient outcomes
8	2022	Tome et al. [15]	Brazil	32262 patients	A single-center prospective study	KDIGO criteria	The EMR system	The 30-day mortality in patients with AKI was reduced
9	2023	Kotwal et al. [16]	Australia	639 patients	A prospective cohort study	KDIGO criteria	The EMR system	The length of stay for patients with AKI was predominantly reduced, while improvements were observed in AKI documentation, nephrology consultation rates, and discontinuation of nephrotoxic medications
10	2024	Nada et al. [17]	USA	2883 newborns	A quality improvement project	SCr level >1.5 mg/dL	The EMR system	Integration of an EMR alert system with automated documentation offered an effcient and economical solution for improving neonatal AKI diagnosis and documentation

**Table 3:** New biomarkers.

New biomarkers	Significance	Advantages	Disadvantages	Feasibility	Application case in electronic early warningsystems
CysC	CysC is a small molecular protein that can be freely filtered through the glomeruli and completely reabsorbed in the proximal tubules. It is almost undetectable in urine. CysC is an endogenous marker of glomerular filtration rate	CysC was not associated with age, sex, inflammation, or muscle mass.It is an early, sensitive and specific predictor of AKI occurrence and an important prognostic indicator for AKI patients	There are some interfering factors such as glucocorticoid therapy and inflammation, which can increase serum cystatin C levels. Cystatin C is limited in the diagnosis of early acute kidney injury due to the specific disease process and therapeutic environment	Blood can be drawn to obtain results	As of now, no relevant research has been found
KIM-1	KIM-1 is a type I transmembrane glycoprotein, which can be used in the differential diagnosis of glomerular and renal tubule injury. It is almost not expressed in normal tissues, but is rapidly up-regulated in proximal tubule epithelial cells when ischemic or toxic injury occurs	Urinary KIM-1 can be used as an early biomarker of acute tubular necrosis and predict adverse clinical outcomes such as dialysis and death in patients with AKI	It may also be elevated due to drug toxicity, infection, or autoimmune disease	Blood can be drawn to obtain result	As of now, no relevant research has been found
NGAL	NGAL is mainly expressed in the proximal tubules. During acute renal injuries, renal tubule epithelial cells are stimulated by injury, and a large amount of NGAL is secreted	Levels of NGAL in urine and blood can increase significantly within 2 hours after renal injury, making NGAL a valuable prognostic indicator for AKI	Some extrarenal diseases may affect its concentration and thus make it less specific	Blood and urine can be drawn to obtain results	As of now, no relevant research has been found
TIMP2 and IGFBP7	TIMP-2 and IGFBP7 act as part of the cell cycle checkpoint. Both are soluble proteins produced by the kidneys and are thought to play a role in G1 cell cycle arrest during early stages of cellular stress and renal tubular injury.The combination of TIMP-2 and IGFBP7 can enhance diagnostic accuracy, and urinary TIMP-2 • IGFBP7 serves as a reliable predictor for the onset of acute kidney injury	Not affected by other chronic diseases or acute diseases other than AKI	In some studies, it has been found that (TIMP-2) × (IGFBP-7) only performs well in predicting moderate to severe AKI, but performs poorly in predicting mild AKI	It can be obtained from urine	The study conducted by Chiara Levante et al. provides insights for clinical practice: when a Nephrocheck Test result is >0.3 ng/dL, an automatic electronic alert prompts physicians to intervene through preventive measures
RONS	RONS is a biomarker for the early AKI process	Accurate detection of renal RONS can facilitate early diagnosis of AKI	There is relatively little research on this topic	Multiple methods can be used to detect	As of now, no relevant research has been found

## Data Availability

No data were used to support the findings of this study.
